# What do language barriers cost? An exploratory study among asylum seekers in Switzerland

**DOI:** 10.1186/1472-6963-10-248

**Published:** 2010-08-23

**Authors:** Alexander Bischoff, Kris Denhaerynck

**Affiliations:** 1 Institute of Nursing Science, Faculty of Medicine, University of Basel, Basel, Switzerland; 2 Department of Community Medicine and Primary Care, Division of International and Humanitarian Medicine, Geneva University Hospitals, Geneva, Switzerland

## Abstract

**Background:**

Language barriers have a major impact on both the quality and the costs of health care. While there is a growing body of evidence demonstrating the detrimental effects of language barriers on the quality of health care provision, less is known about their impact on costs. This purpose of this study was to investigate the association between language barriers and the costs of health care.

**Methods:**

The data source was a representative set of asylum seekers whose health care was provided by a Swiss Health Maintenance Organisation (HMO). A cross-sectional survey was conducted: data was collected on all the asylum seekers' health care costs including consultations, diagnostic examinations, medical interventions, stays in the clinic, medication, and interpreter services. The data were analysed using path analysis.

**Results:**

Asylum seekers showed higher health care costs if there were language barriers between them and the health professionals. Most of these increased costs were attributable to those patients who received interpreter services: they used more health care services and more material. However, these patients also had a lower number of visits to the HMO than patients who faced language barriers but did not receive interpreter services.

**Conclusion:**

Language barriers impact health care costs. In line with the limited literature, the results of this study seem to show that interpreter services lead to more targeted health care, concentrating higher health care utilisation into a smaller number of visits. Although the initial costs are higher, it can be posited that the use of interpreter services prevents the escalation of long-term costs. A future study specially designed to examine this presumption is needed.

## Background

Language barriers have a major impact on both the quality and costs of health care provision. This is increasingly important in this age of global migration [1]. One result of global migration is that health care providers face a diverse range of patients with whom they have no language in common. At the same time they are required to provide high quality health care to these patients in line with the principles of human rights and equity.

Unequal treatment related to language barriers is associated with unequal access to health care and unequal health outcomes [2]. For example, a recent study by Ou et al [3] confirmed that patients who do not speak the local language were disadvantaged in their access to health services. Language barriers also result in unequal treatment. Patients who face language barriers have poorer health outcomes when compared with patients who are proficient in the local language [4-7].

A growing body of evidence documents the different ways in which language barriers impact on the quality of care. Where there are language barriers, patient-provider communication tends to be less successful [8], patient satisfaction is reduced [9] and provider dissatisfaction is increased [10]. Patients facing language barriers are also likely to consume more health care [10,11], and to have more frequent adverse events [6,12,13]. In one recent study, Divi reviewed serious medical errors in six US hospitals and found that adverse events occurred far more frequently in relation to patients who spoke little English than to those who were proficient in English. She concludes: "Language barriers appear to increase the risks to patient safety" [6:60].

In contrast, the use of professional interpreters is associated with better quality care [14,15] and has been shown to reduce disparities [14,16,17]. Studies suggest that foreign-language patients who have access to professional interpreters have improved outcomes, for example, less hospitalisation, better chronic disease outcomes and lower health care costs [10,17-21]. In a retrospective cohort study in a large HMO (Health Maintenance Organisation), Jacobs et al showed the effectiveness of professional interpreter services in improving the delivery of health care to a population of foreign-language speakers [22]. Patients who used the interpreter services had a significantly higher number of consultations, prescriptions and screening examinations than the control group. With this increased access came higher costs. These were counterbalanced, however, by a lower number of referrals and increased provision of ambulant and preventive care [23,24]. Jacobs et al argue that the cost of providing interpreters is minimal compared to the costs of managing chronic diseases such as diabetes or cardio-vascular disease. They conclude that providing interpreter services improves foreign-language patients' access to care and that this "is a financially viable method for enhancing delivery of health care to patients with limited English proficiency" [23].

In order to add to the knowledge base in this field of research, we investigated the associations between language barriers, the costs of health care, and access to health care for a group of asylum seekers.

## Methods

### Design, sample and setting

This cross-sectional study used a representative sample of asylum seekers in Switzerland.

People seeking protection in Switzerland can file an asylum application at one of the five border-crossing reception centres of the Federal Office for Migration. There they undergo a first brief round of questioning about their reasons for seeking asylum and have a health check. This includes screening for tuberculosis and hepatitis B as well as immunisation. The Swiss border-crossing health check is comparable to the screening checks provided for asylum seekers in most other European countries [25]. Asylum seekers are then allocated to one of the 26 Swiss cantons in line with a pre-established quota system. Primary health care for asylum seekers is provided by the public health system in each canton. Since health care insurance is mandatory for all those living on Swiss territory [26], asylum seekers are provided with insurance by the Federal Office of Migration for as long as they have asylum seeker status [27]. The insurance includes free access to health care and coverage of all health care costs. In some cantons, health care and health insurance schemes are organised by specialised Health Maintenance Organisations (HMO).

We studied the full insurance costs of a sample of asylum seekers in a major canton which, under a pre-set federal quota system, took in 2.3% of asylum seekers in Switzerland. The asylum seekers assigned to this canton were distributed alternately to two Health Maintenance Organisations (HMO) [28], one of which, the A-care HMO, was set up for this purpose (A stands for asylum). All the asylum seekers attending this HMO were included in this study. The A-care HMO was integrated within the Department of Ambulatory Internal Medicine at the cantonal University Hospital [29]. Care was coordinated by the hospital's Department of Ambulatory Internal Medicine which provided primary care to about 70% of the HMO's patients.

### Data collection

The data for this study were routinely collected by the hospital administration and merged with additional demographics obtained from the Federal Office for Migration. The data were anonymised. The study received clearance from the ethical committee (Ethik-Kommission beider Basel) and spanned a period from the start of the A-care program at the beginning of January 2000 through the end of December 2003, after which the program was terminated. Patients had free access to their HMO. All health care costs were completely covered by the HMO, including those not generated within the outpatient clinic.

### Variables and measurements

Data on costs were collected by the accounting office of the hospital administration and reflected consultations, diagnostic examinations (lab, x-rays, ECG, MRI etc.), medical interventions, patients' stays in the clinic, and medication. Costs relating to professional interpreters are included in the costed items for clinic visits and are part of the "package" for asylum seekers. The costs are expressed per month and have been converted from Swiss Francs into Euros using the exchange rate at December 31, 2002 (1 Swiss Franc = 0.68795 EUR; cf. http://www.oanda.com).

Data about language barriers were extracted from the patient records. We distinguished three categories: a) no reported language barriers between asylum seeker and physician, b) reported language barriers between asylum seeker and physician with the provision of interpreter services, and c) reported language barriers between asylum seeker and physician but no provision of interpreter services.

It was not mandatory for physicians to record language barriers in the patients' files. Language barriers were only reported, therefore, when the asylum seekers had serious health conditions and detailed communication was essential. The language proficiency of the physicians was not recorded but it is known that they were able to speak German, English, French, and Italian.

Other variables for this study included gender, age, the number of visits to the HMO, and the number of diagnoses. For the latter, the study used the eight categories of diagnostic groups in the ICD system (diseases of the musculoskeletal system, the respiratory system, mental disorders, skin diseases, injuries, infectious and parasitic diseases, pregnancy-childbirth-puerperium, and cardiovascular diseases) [30]. The patients' utilisation of health care services and material was also assessed. This consisted of the sum of all the medical material used, the medications prescribed, and medical/nursing interventions.

### Data analysis

**Figure 1:**
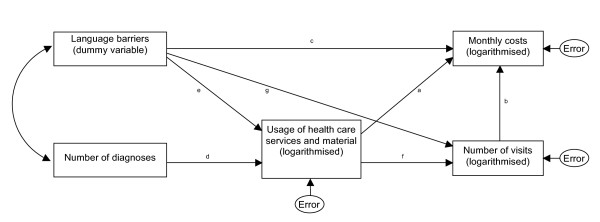
**Theoretical framework used in the path analysis**.

Analysis of variance with multiple comparisons (Tukey test) was used to compare costs among the three language barrier categories. Further, we hypothesised that health care costs would reflect the patients' usage of health care services and material as well as the number of visits at the HMO, and that health care usage would also be a function of the number of diagnoses that a patient had (representing their health status). Figure 1 shows the assumed relationships in a model that we tested using path analysis. The effects of language barriers on costs, health care usage and visits was examined by dummy coding the three categories (a. individuals facing language barriers and using an interpreter, b. facing language barriers but not using an interpreter, and c. facing no language barriers). Path analysis uses multiple regression analysis techniques to test complex and pre-specified relationships between the predictor (exogenous) and outcome (endogenous) variables. We entered "language barriers" and "number of diagnoses" as exogenous variables, and the logarithmically transformed variables "health care costs", "health care usage" and "number of visits" as endogenous, and fitted the model using the statistical program SAS 9.1.3. [31].

## Results

Of the 795 asylum-seeker patients enrolled in the A-Care programme who visited the service at least once, information about whether they faced language barriers was available for 486 (61.1%).

These 486 asylum seekers came from the following countries or regions: 50% from the Balkans (Serbia-Montenegro, Kosovo, Bosnia-Herzegovina); 11% from sub-Saharan Africa (including, in decreasing order, people from the Congo, Angola, Nigeria, Togo, Ethiopia, Cameroun, Guinea, Somalia, Sierra Leone, Liberia, Sudan, Burundi, Mauritania, Guinea-Bissau, Gambia, Côte-d'Ivoire, Kenya, Niger and Burkina Faso); 6% from Turkey; 5% from Iraq; 5% from Sri Lanka; and 23% from other countries (including people from the Afghanistan, Albania, Algeria, Armenia, Azerbaijan, Bangladesh, Belarus, Bulgaria, the Republic of China, Ecuador, Georgia, India, Iran, Kazakhstan, Colombia, Lebanon, Libya, Lithuania, Morocco, Moldavia, Mongolia, Pakistan, Russia, Syria, Tunisia, Ukraine and Vietnam).

Table 1 presents an overview of the sample, comparing asylum seekers who faced language barriers (n = 87; 18%) with those who did not face language barriers. It shows that those facing a language barrier were older and that there were more females in the language barriers group than in the group without language barriers. Patients with language barriers also had two to three times as many ICD diagnoses as patients in the group without reported language barriers. The length of time for which they were insured, their number of visits to the HMO, their consumption of health care, and their monthly health care costs were also two to three times higher than for the group of patients without language barriers (Table 1).

**Table 1:** Sample characteristics of asylum seekers

Variable	No language barrier (n = 399)	Language barrier (n = 87)	p-value (*)
Age (Median, IQR)	25.4 (12.6)	31.9 (14.9)	<.0001
Number of men (Percentage)	264 (66%)	36 (41%)	<.0001
Number of diagnoses (Median, IQR)	1 (1)	2 (2)	<.0001
Duration of A-Care insurance in days (Median, IQR)	273.5 (518)	607 (790)	<.0001
Monthly cost in Euros (Median, IQR)	1278 (2715)	3195.5 (3474)	<.0001
Number of visits per year (Median, IQR)	10.8 (17.8)	23 (19.7)	<.0001
Health care usage per year (Median, IQR)	0.17 (0.28)	0.36 (0.44)	<.0001

(*) Mann-Whitney U test; Chi^2 ^test

The fact that an interpreter had been used was recorded in the medical files of 64 of the 87 asylum-seeking patients who faced language barriers (73.6%; Table 2). Professional interpreters (from the interpreter service) were provided for 39 (60.9%) of these patients, and ad hoc interpreters (including patients' relatives and acquaintances and hospital employees) were used for the other 25 (39.1%).

**Table 2:** Language barriers among asylum seekers (n = 87)

Language barrier specifications	Frequency	Percentage
Language barriers reported, no interpreter present	23	26.4%
Language barriers reported, interpreter present	64	73.6%

Professional interpreter	39	60.9%
Ad hoc interpreter *	25	39.1%

* Ad hoc interpreter includes patient's relatives, patient's acquaintances, and hospital employees

Analysis of variance revealed cost differences between individuals who received an interpreter service (who had higher costs) and those who had no language barriers (who had lower costs; p < 0.0001). Path analysis results are presented in Table 3. The model fit was good (Model Chi^2 ^= 0.80; df = 2; p = 0.67). Health care costs were mainly determined by the consumption of health care services and material (p < .0001) and by the number of visits (p < .0001). Altogether, these explained about 35% of the variability in costs. More specifically, estimates showed that a 10% increase in the consumption of health care services and material was associated with a 2% increase in monthly costs (= 1.10^0.2489^), and that a 10% increase in the number of visits corresponded to a 5% increase in monthly costs (= 1.10^0.562^).

**Table 3:** Results of the path analysis

Outcome variable	R^2^	Predictor variable	Figure	Estimate (=β)	Standard Error	t-value	p-value	**e**^**β**^
Monthly costs (*)	35%	Health care usage (*)	a	0.2489	0.0512	4.8576	<.0001	
		
		Number of visits (*)	b	0.5620	0.0674	8.3437	<.0001	
		
		Language barriers: contrast between categories	c					
		How much more did individuals facing language barriers using an interpreter (1) cost compared to those facing language barriers but not using an interpreter (0)		0.0884	0.2785	0.3175	0.7509	1.09
		How much more did individuals using an interpreter (1) cost compared to those without language barriers (0)		0.1949	0.1650	1.1810	0.2376	1.22
		How much more did individuals with language barriers who did not use an interpreter (1) cost compared to those without language barriers (0)		0.1065	0.2450	0.4345	0.6640	1.11

Usage of health care services and material (*)	41%	Number of ICD diagnoses	d	0.4293	0.0286	14.9971	<.0001	
		
		Language barriers: contrast between categories	e					
		How much more health care did individuals facing language barriers using an interpreter (1) consume compared to those with language barriers but not using an interpreter (0)		0.6292	0.2538	2.4794	0.0132	1.88
		How much more health care did individuals using an interpreter (1) consume compared to those without language barriers (0)		1.0195	0.1443	7.0637	<.0001	2.77
		How much more health care did individuals with language barriers who did not receive interpreter services (1) consume compared to those without language barriers (0)		0.3902	0.2240	1.7418	0.0815	1.48

Number of visits (*)	40%	Health care usage (*)	f	0.4604	0.0276	16.6640	<.0001	
		
		Language barriers: contrast between categories	g					
		How many more visits did individuals with language barriers using an interpreter (1) pay to the HMO compared to those with language barriers but not using an interpreter (0)		-0.2838	0.1877	-1.5122	0.1305	0.75
		How many more visits did individuals using an interpreter (1) pay to the HMO compared to those without language barriers (0)		-0.0412	0.1114	-0.3696	0.7117	0.96
		How many more visits did individuals with language barriers who did not use an interpreter (1) pay to the HMO compared to those without language barriers (0)		0.2426	0.1652	1.4690	0.1418	1.27

Explanation to the table: Outcome variables of the model are presented in the first column. Explanatory variables are listed in column three. The column in between (R^2^) represents the proportion of variability explained in a particular outcome variable by its set of predictor variables. Each of the relationships between predictor and outcome variables are indicated by the letters 'a' to 'g', which refer to their related arrows in Figure 1.

Language barriers are the variables of primary interest. Since these were dummy coded, the regression coefficients in column 5 represent increases in the value of the outcome variable for each 1-unit increase in the dummy variable, i.e., the increase for the language barrier category indicated with (1) as compared to the reference category indicated with (0). For instance, comparing individuals facing language barriers using an interpreter (1) to individuals facing language barriers but not using an interpreter (0) shows that the former are associated with a monthly cost score that is 0.0884 times higher than the reference. Note that monthly costs, as all variables indicated with an asterisk (*) have been logarithmically transformed to a normal distribution, which can conveniently be interpreted in terms of relative changes presented in the last column. Patients using an interpreter generated exp(0.0884) = 9% more monthly costs compared to those facing language barriers but not using an interpreter.

Language barriers were not independently related to costs (despite the fact that fees for professional interpreters were part of the cost variable). They were, however, indirectly related, as asylum seekers who had an interpreter consumed 2.8 times more health care services and material than those who did not face language barriers (p < .0001), and 88% more health care services and material than those who faced language barriers but had no interpreter (p = 0.01). Although health care consumption was higher when interpreters were used, the number of visits by this group was only 75% of the number of visits by patients who faced language barriers but did not receive interpreter services. However, this trend did not reach significance (p = 0.13).

To prevent inference from small subsamples, we did not differentiate between professional and ad hoc interpreters in our path model. However, as an exploratory analysis, we examined the possible effects of the two types of interpreting by testing the model first with only professional interpreters, excluding the ad hoc interpreters, and then with only ad hoc interpreters, excluding the professional interpreters, leaving all other parameters equal. The sub-model with only professional interpreters, excluding the ad hoc interpreters was more or less similar to the grand model of table 3. The model excluding the professional interpreters differed from the grand model, in that table 3 shows a significantly higher health care consumption in individuals facing language barriers without interpreter compared to those with an interpreter (p = 0.01; 88%), whereas in the new model, this difference disappeared (p = 0.20; 48%), suggesting that the presence of professional interpreters affected health care consumption more than the presence of ad hoc interpreters.

## Discussion

This study is one of a few that attempt to quantify the costs associated with language barriers. Language barriers were identified in a sample of asylum seekers. The extent to which they influenced costs, either directly or indirectly (via known cost drivers, e.g., consumption of health care services, number of visits) was measured. Uncontrolled comparisons showed that costs were twice as high for asylum seekers who faced language barriers than for those who did not.

However, no direct link was found when other major cost-generating factors were controlled for using a path-analytic model. Instead, the variable "consumption of health care services and material" served as a moderator variable, showing that asylum seekers who used an interpreter received more health care and, as a consequence, cost the system more. This finding is in line with the study by Jacobs et al who also found increases in health care usage once interpreter services were introduced [22].

In line with another study by Jacobs et al. [23] we found that, although patients who used interpreters consumed more health care, they made a lower number of visits to the HMO than patients facing language barriers who did not use interpreters. The relationship was not significant in our study (probably due to the small number of patients in this group). However, it is in line with evidence suggesting that referrals are much better targeted if the health care provider has a clear understanding of a patient's condition [32]. In other words, the presence of an interpreter makes it possible to reach an effective solution after fewer visits. And although the resulting increased provision of health care drives costs up in the short term, it is very likely that suboptimal problem-solving across language barriers without an interpreter leads to increased costs in the longer term because the patient's health problems are unlikely to be resolved. The present study was unable to determine whether the higher costs associated with having an interpreter pay off in the long term because it was limited in time.

A potential limitation of our study is that language barriers may only have become apparent and been recorded for those patients who needed the most care, resulting in a large group of relatively healthy patients for whom no reliable data on language proficiency are available. The observation that those patients not recorded as facing language barriers were the least expensive may represent a bias in the measurement of language proficiency. The fact that we found a higher number of diagnoses in the language barrier group than in the no language barrier group suggests that this possibility is plausible. In fact, many of the differences between patients facing language barriers and those not in the bi-variate comparisons of Table 1 may be a reflection of this bias. Alternatively, it is also possible that the combination of one or more serious conditions (chronic diseases) and language barriers reinforced each other. In other words, the fact that the patients facing language barriers generated more health care usage and costs may have been, in some ways and to some extent, due to the language barriers rather than to prior medical conditions. Despite the possibility of measurement bias in the no-barriers category, our findings are in line with Jacobs et al whose study setup was less prone to this kind of bias [22-24]. The limitation in our study can be overcome in the future by systematically measuring language barriers and also by including patients' views as to whether they faced language barriers in their communication with health care providers. Other limitations of our study included the lack of socio-economic data about the asylum seekers that could be related to the degree of language barriers, the lack of knowledge of the language proficiencies of the physicians, and the small number of interpreters involved in the A-care programme. It is possible that increased use of professional interpreters would increase the effect sizes, as suggested by the fact that our exploratory analysis only found higher health care usage if ad hoc interpreters were omitted from analysis. Nevertheless, even the use of ad hoc interpreters seems already to affect the health care process.

These limitations apart, this study has several strengths. First, the patient cohort, i.e., the asylum seekers, with their trajectory of migration or even exile, are a population at risk and one for which linguistic assistance is of particular importance [2]. Second, while most other related studies included general average patient costs only (apart from the costs of interpreters) [22,24,33], this study was able to measure all the specific individual costs generated by each asylum seeker's use of health care. Third, we were able to put the relevant variables that emerged from the limited literature on language barriers and costs into one model, thereby corroborating the existing hypotheses.

## Conclusion

This exploratory study provides useful information in an area where there is a serious lack of data, i.e., language barriers in health care and their relationship to costs. The results suggest that language barriers can no longer be ignored in mainstream health care services since they may generate considerable long-term costs if left unaddressed. Scientifically sound data are needed on the impact of language barriers and of providing interpreters on the quality of care and on the resulting costs [16]. Further investigation of the cost-effectiveness of interpreter services is required. This could take the form, for example, of a trial in which professional interpreters, ad hoc interpreters, and no interpreters are randomly assigned to people screened for language barriers who are enrolled in a given HMO. We are fully aware that costs are not the only thing that matters, but, at a time of increasing pressure on health care costs, we deemed it important and useful to consider the economic arguments for and against the use of interpreters as part of a portfolio of measures to reduce health care inequalities.

## Competing interests

The authors declare that they have no competing interests.

## Authors' contributions

AB conceptualised the aggregation of the databases, provided the study design and drafted the manuscript. KD carried out the analyses and revised the manuscript critically for intellectual content. AB and KD have given final approval of the version to be published.

## Pre-publication history

The pre-publication history for this paper can be accessed here:

http://www.biomedcentral.com/1472-6963/10/248/prepub

## References

[B1] Castles S Miller MJ The age of migration: international population movements in the modern world 1993 Houndmills [etc.]: MacMillan

[B2] Messias DKH McDowell L Estrada RD Language Interpreting as Social Justice Work: Perspectives of Formal and Informal Healthcare Interpreters Advances in Nursing Science 2009 32 2 128 143 110.1097/ANS.1090b1013e3181a1093af1097 19461230 10.1097/ANS.0b013e3181a3af97

[B3] Ou L Chen J Hillman K Health services utilisation disparities between English speaking and non-English speaking background Australian infants BMC Public Health 2010 10 182 10.1186/1471-2458-10-182 20374663 PMC2858120

[B4] Fiscella K Franks P Doescher MP Saver BG Disparities in health care by race, ethnicity, and language among the insured: findings from a national sample Med Care 2002 40 1 52 59 10.1097/00005650-200201000-00007 11748426

[B5] Flores G Laws MB Mayo SJ Zuckerman B Abreu M Medina L Hardt EJ Errors in medical interpretation and their potential clinical consequences in pediatric encounters Pediatrics 2003 111 1 6 14 10.1542/peds.111.1.6 12509547

[B6] Divi C Koss RG Schmaltz SP Loeb JM Language proficiency and adverse events in US hospitals: a pilot study Int J Qual Health Care 2007 19 2 60 67 10.1093/intqhc/mzl069 17277013

[B7] Derose KP Baker DW Limited English proficiency and Latinos' use of physician services Med Care Res Rev 2000 57 1 76 91 10.1177/107755870005700105 10705703

[B8] Rivadeneyra R Elderkin-Thompson V Silver RC Waitzkin H Patient centeredness in medical encounters requiring an interpreter Am J Med 2000 108 6 470 474 10.1016/S0002-9343(99)00445-3 10781779

[B9] Carrasquillo O Orav EJ Brennan TA Burstin HR Impact of language barriers on patient satisfaction in an emergency department J Gen Intern Med 1999 14 2 82 87 10.1046/j.1525-1497.1999.00293.x 10051778

[B10] Hampers LC McNulty JE Professional interpreters and bilingual physicians in a pediatric emergency department: effect on resource utilization Archives of Pediatrics & Adolescent Medicine 2002 156 11 1108 1113 10.1001/archpedi.156.11.1108 12413338

[B11] Smedley BD Stith AY Nelson AR Unequal treatment. Confronting racial and ethnic disparities in health care 2003 Washington: The National Academies Press 25032386

[B12] Cohen AL Rivara F Marcuse EK McPhillips H Davis R Are Language Barriers Associated With Serious Medical Events in Hospitalized Pediatric Patients? Pediatrics 2005 116 3 575 579 10.1542/peds.2005-0521 16140695

[B13] Bartlett G Blais R Tamblyn R Clermont RJ MacGibbon B Impact of patient communication problems on the risk of preventable adverse events in acute care settings Cmaj 2008 178 12 1555 1562 18519903 10.1503/cmaj.070690 PMC2396356

[B14] Karliner LS Jacobs EA Chen AH Mutha S Do professional interpreters improve clinical care for patients with limited english proficiency? A systematic review of the literature Health Serv Res 2007 42 2 727 754 10.1111/j.1475-6773.2006.00629.x 17362215 PMC1955368

[B15] Morales LS Elliott M Weech-Maldonado R Hays RD The impact of interpreters on parents' experiences with ambulatory care for their children Med Care Res Rev 2006 63 1 110 128 10.1177/1077558705283125 16686075 PMC1634763

[B16] Jacobs E Chen AH Karliner LS Agger-Gupta N Mutha S The need for more research on language barriers in health care: a proposed research agenda Milbank Q 2006 84 1 111 133 10.1111/j.1468-0009.2006.00440.x 16529570 PMC2690153

[B17] Flores G The impact of medical interpreter services on the quality of health care: a systematic review Med Care Res Rev 2005 62 3 255 299 10.1177/1077558705275416 15894705

[B18] Baker DW Parker RM Williams MV Coates WC Pitkin K Use and effectiveness of interpreters in an emergency department JAMA 1996 275 10 783 788 10.1001/jama.275.10.783 8598595

[B19] Graham EA Jacobs TA Kwan-Gett TS Cover J Health services utilization by low-income limited English proficient adults J Immigr Minor Health 2008 10 3 207 217 10.1007/s10903-007-9069-3 17687651

[B20] Chan YF Alagappan K Rella J Bentley S Soto-Greene M Martin M Interpreter services in emergency medicine J Emerg Med 2008 doi:10.1016/j.jemermed.2007.09.045 10.1016/j.jemermed.2007.09.045 18571358

[B21] Fernandez A Schenker Y Time to establish national standards and certification for health care interpreters Patient Educ Couns 2010 78 2 139 140 10.1016/j.pec.2009.12.008 20117487

[B22] Jacobs EA Lauderdale DS Meltzer DO Shorey JM Levinson W Thisted RA The impact of interpreter services on delivery of health care to limited English proficient patients J Gen Intern Med 2001 16 468 474 10.1046/j.1525-1497.2001.016007468.x 11520385 PMC1495243

[B23] Jacobs EA Shepard DS Suaya JA Stone EL Overcoming language barriers in health care: costs and benefits of interpreter services Am J Public Health 2004 94 5 866 869 10.2105/AJPH.94.5.866 15117713 PMC1448350

[B24] Jacobs EA Sadowski LS Rathouz PJ The impact of an enhanced interpreter service intervention on hospital costs and patient satisfaction J Gen Intern Med 2007 22 Suppl 2 306 311 10.1007/s11606-007-0357-3 17957416 PMC2078550

[B25] Norredam M Mygind A Krasnik A Access to health care for asylum seekers in the European Union--a comparative study of country policies Eur J Public Health 2006 16 3 286 290 10.1093/eurpub/cki191 16230318

[B26] OECD OECD Reviews of Health Systems Switzerland 2006 Paris: WHO & OECD

[B27] Wieland T Gesundheitsversorgung von Asylsuchenden: Grenzen unseres Gesundheitssystems Schweizerische Aerztezeitung 2000 81 2673 2676

[B28] Reinhardt UE The Swiss health system: regulated competition without managed care Jama 2004 292 10 1227 1231 10.1001/jama.292.10.1227 15353536

[B29] Bischoff A Schneider M Denhaerynck K Battegay E Health and ill health of asylum seekers in Switzerland: an epidemiological study Eur J Public Health 2009 19 1 59 64 10.1093/eurpub/ckn113 19158102

[B30] DIMDI ICD-10. Internationale statistische Klassifikation der Krankheiten und verwandter Gesundheitsprobleme. Band 1 2001 Bern: Hans Huber

[B31] Hatcher L A step-by-step approach to using the SAS system for factor analysis and structural equation modeling 1994 Cary NS: SAS Publishing

[B32] Bischoff A Bovier P Rrustemi I Gariazzo F Eytan A Loutan L Language barriers between nurses and asylum seekers: their impact on symptom reporting and referral rates Soc Sci Med 2003 57 503 512 10.1016/S0277-9536(02)00376-3 12791492

[B33] Goldsmith C Slack-Smith L Davies G Dentist-patient communication in the multilingual dental setting Australian Dental Journal 2005 50 4 235 241 10.1111/j.1834-7819.2005.tb00366.x 17016888

